# Construction of Promoter Elements for Strong, Moderate, and Weak Gene Expression in *Drosophila melanogaster*

**DOI:** 10.3390/genes16010003

**Published:** 2024-12-24

**Authors:** Ksenia S. Kudryashova, Irina O. Deriglazova, Igor S. Osadchiy, Pavel Georgiev, Oksana Maksimenko

**Affiliations:** 1 Center for Precision Genome Editing and Genetic Technologies for Biomedicine, Institute of Gene Biology, Russian Academy of Sciences, 34/5 Vavilova Str., Moscow 119334, Russia; ksebiona@gmail.com (K.S.K.); deriglazovai@mail.ru (I.O.D.);; 2 Department of the Control of Genetic Processes, Institute of Gene Biology, Russian Academy of Sciences, 34/5 Vavilova Str., Moscow 119334, Russia; georgiev_p@mail.ru

**Keywords:** transgene, protein expression, transcription activation, regulatory elements

## Abstract

Background/Objectives: Transcriptional promoters play an essential role in regulating protein expression. Promoters with weak activity generally lead to low levels of expression, resulting in fewer proteins being produced. At the same time, strong promoters are commonly used in studies using transgenic organisms as model systems. This approach can have various negative consequences for the organism, as many regulatory proteins need to be expressed in small quantities, and excessive expression can have harmful effects on cells and organisms. Therefore, it is important to select the right promoter when creating transgenic organisms for research and practical applications. Methods: In this study, we used the *Drosophila melanogaster* genome as a source of natural promoter sequences for RNA polymerase II. These sequences were extracted and used to create a set of promoters that are suitable for practical application. The promoters were tested in a model system using fluorescent reporter genes in S2 cells and transgenic lines of *Drosophila*. Results: We assessed the expression levels of fluorescent reporter genes to rank the tested promoters from strongest to weakest. Six individual promoters of different sizes were established and compared. Additionally, we designed and tested three pairs of bidirectional promoters that could be used to simultaneously express two proteins. Conclusions: Based on our findings, we grouped the tested promoters into three categories: strong, moderate, and weak. These promoters can be utilized in transgenic model systems for protein production at different levels, from high to low. Bidirectional promoters, constructed “head-to-head”, meaning oppositely directed with the minimum distance between them, represent a novel tool for the co-expression of proteins.

## 1. Introduction

*Drosophila melanogaster* (*D. melanogaster*) remains one of the most convenient model organisms for studying gene expression and transcription regulation. *Drosophila* genes and their products, as well as accumulated knowledge and advanced genome editing techniques, facilitate the understanding of how genes function and which regulatory regions and transcription factors control this process. Coordination of the expression of genes in time and space is essential for proper organism development and function.

From an experimental perspective, studying genes and proteins often involves modifying them in order to investigate structure–function relationships. To this end, mutated genes or tagged proteins are expressed in transgenic flies or cells for analysis. In the case of endogenous expression under a native promoter, the optimal level of gene product is achieved via the native set of regulatory elements. If this is not possible or necessary, ectopic expression under a native promoter can be used, although it requires additional testing of promoter activity outside the local environment. The extracted promoter sequence chosen for construct development may vary in size and thus contain fewer or more regulatory elements. Commonly, genes are expressed exogenously under the control of ubiquitous promoters such as those of *actin 5C* [1] or *heat shock protein 70* (*hsp70*) [2]. However, even these commonly used promoters have limitations. Strong expression from the *hsp70* promoter requires heat induction, which may have negative effects on long-term studies [3] while the *actin 5C* promoter has a certain developmental specificity [4].

Despite the obvious advantages of using strong ubiquitous promoters, such as increased productivity and availability of plasmids, a high level of gene expression may not always be necessary and can even be harmful. Although many proteins do not interfere with the normal development and function of the cells, the inappropriate expression of transcription factors and regulatory molecules typically has harmful effects [5]. Elevated protein concentrations can alter normal organism development, leading to lethality and ultimately affecting the reliability of data obtained. According to some estimates, approximately 60% of *Drosophila* genes encode proteins that can cause problems if they are mis- or overexpressed [6]. For example, the transcription factor Grainy head is expressed in the developing trachea and is required to prevent excessive tube extension; however, its overexpression in all tracheal cells can limit lumen growth [7]. The transcription factor zinc-finger homeodomain 1 (Zfh1) is an important regulator of the immune response in *Drosophila*, but its overexpression blocks the innate immunity pathway in *Drosophila* S2 cells [8]. Overexpression of the transcriptional regulator Dorsal switch protein 1 (DSP1) in the adult *Drosophila* brain induces neuronal dysfunction and neurodegeneration phenotypes [9].

For proteins that need to be expressed at constitutively low levels in cells, it can be useful to use promoters with reduced activity. In contrast to strong promoters, there is little information in the literature about moderate and weak promoters, at least for *Drosophila*, and selecting the right promoter may be challenging. It should be noted that, in addition to transcriptional regulation, other key steps in protein synthesis may also be affected in order to achieve an appropriate protein level. In cap-dependent translation, the efficient recognition of the AUG initiation codon by the small ribosomal subunit of the preinitiation complex depends on its surrounding Kozak sequence and constitutes one of the main mechanisms of translation regulation. The optimal context for AUG recognition in eukaryotes has been proposed to be the consensus CRCCaugG (where R represents a purine, i.e., A or G) [10]. However, in *Drosophila* many mRNAs have different Kozak sequences that are predicted to be weaker [11]. Acevedo and co-authors systematically analyzed how translation rates depend on the Kozak sequence in *Drosophila* and created a useful tool for predicting translation strength based on the Kozak sequence [11]. We used this resource to create a construct with an attenuated Kozak sequence that leads to a reduced level of protein expression. Regulation of protein production at a translation level may be applicable if transcriptional regulation is not feasible or if minimal interference with the genome is required.

Finally, an intriguing question arises: can two promoters be combined into one bidirectional transcription unit? “Bidirectional promoters” are regulatory regions shared between two genes that are arranged on opposite strands, with their 5′ ends close to each other and initiate transcription in opposite directions [12]. In humans, divergently transcribed gene pairs whose transcription start sites are separated by less than 1000 bp represent more than 10% of genes in the genome [13]. The fraction of gene pairs in *D. melanogaster* that are in close proximity is even higher, at 31.6% of all genes [14,15]. For example, gene *cs1* is located just 94 bp upstream of the *ras2* gene, and the *ras2*/*cs1* promoter is one of the shortest bidirectional promoters in *D. melanogaster* [16,17].

Due to their compact design, bidirectional promoters have benefits over single gene expression cassettes. However, their rational design is complicated by unclear regulatory mechanisms and the difficulty in predicting their efficiency. The close proximity between different regulatory elements may cause either cooperation and increased expression via shared elements that regulate both genes, or interference because of competition between promoters for the transcription machinery, or there may be no apparent effects [12]. The mutual influence of bidirectional promoters in *D. melanogaster* remains underexplored.

In this study, we extracted several promoter sequences for RNA polymerase II from the *Drosophila* native genomic environment and inserted them into the same landing platform. We then assessed the expression level of fluorescent reporter genes to rank the promoters from strong to very weak. Six individual promoters of different sizes were established and compared. These promoters can be further used for the production of proteins at different levels, ranging from high to very low. We also designed and tested three pairs of bidirectional promoters that can be used to express two proteins simultaneously. The resulting constructs containing these promoters are already in use by our team and have the potential to be applied to a wide range of applications in other labs working with *Drosophila*.

## 2. Materials and Methods

### 2.1. Transgenic Fly Generation

*Drosophila* strains were grown at 25 °C under standard culture conditions. The constructs were inserted into the fly genome using the φC31-mediated site-specific integration system [18]. The construct-containing plasmids were injected into *y^1^w^1118^* pre-blastoderm embryos (86Fb fly line) bearing an attP site for integration at the 86F8 locus (chromosome III) and *φC31* integrase under the control of the *vasa* promoter (chromosome I). Emerging adults were crossed with *y^1^w^1118^* flies, and the transgenic progeny were identified by their eye color (w+, *white* marker) or pigmented cuticle (y+, *yellow* marker). All obtained lines were studies as homozygotes. Details of the crosses are available upon request.

### 2.2. S2 Cell Line Transfection

*Drosophila* S2 cells were grown in SFX medium (HyClone, Logan, UT, USA) at 25 °C. Transfection of plasmids was performed with the Cellfectin II (Invitrogen, Waltham, MA, USA) or Maxfectin (Miltenyi Biotec, Gaithersburg, MD, USA) reagent according to the manufacturer’s instructions. Typically, cells were transfected in six-well plates and grown for 24–48 h before flow cytometric analysis. All transfection procedures were performed with three independent replicates.

### 2.3. Plasmid Construction

For transfection of S2 cells and for generation of transgenic flies, promoter fragments were PCR-amplified using corresponding primers either from the fly genome (pCG10321, pCP190, pPzg, and pZIPIC) or previously described plasmids (pUbi, mini-pRpL, and pRpL*). For single promoter-containing constructs, PCR-amplified promoter fragments were cloned into the *pBluSK* plasmid with the *yellow* reporter gene, LoxP site, SV40 polyadenylation signal, and attB site. Five Pita-binding sites (Pita×5; Supplementary Table S1) were added 22 bp upstream of the promoter sequence. Part of the *dCTCF* gene comprising the last (third) intron, the last (third) exon and the 3′ untranslated region (3′UTR) (Supplementary Table S1) was inserted 15 bp downstream of the *mCherry* stop codon and at 10 bps upstream of the SV40 polyadenylation signal. PCR-directed mutagenesis was applied to mutate the Kozak sequence in the pUbi plasmid to obtain the pUbi_K plasmid (Supplementary Table S2).

For bidirectional promoters, PCR-amplified promoter fragments were cloned into the *pBluSK* plasmid with the *white* reporter gene, LoxP site, SV40 polyadenylation signal, and attB site, and without Pita×5 sites and the *dCTCF* 3′UTR region. *eGFP* and *mCherry* protein-coding sequences were PCR-amplified and cloned under corresponding promoters in each plasmid. Plasmid maps and cloning details are available on request.

### 2.4. Fluorescence and Confocal Microscopy

Collected embryos were dechorionated, mounted on a cover glass via a heptane glue, embedded in Halocarbon 27 oil (Sigma, Livonia, MI, USA), and covered with a second cover glass. All confocal images were acquired with a Leica STELLARIS 5 confocal microscope using a Leica HC PL APO 40×/1.30 oil immersion objective. eGFP fluorescence was excited with a 488 nm laser, and emission was registered within the 500–550 nm range. mCherry fluorescence was excited with a 543 nm laser, and emission was registered within the 550–650 nm range. Third-instar larvae were immobilized in 10% glucose by freezing, placed on a slide glass and imaged on a Nikon Eclipse Ti fluorescence microscope with a Nikon Plan Fluor 4×/0.13 objective (Nikon Instruments Inc., Melville, NY, USA).

### 2.5. Flow Cytometry

Cells were washed with PBS twice and analyzed using a MACSQuant Analyzer 10 (Miltenyi Biotec, San Diego, CA, USA) flow cytometer. Untransfected cells served as negative control and were used for gating. All parameters of the measurements that could affect the intensity of the detected signal were fixed.

### 2.6. Image Processing and Data Analysis

All image processing was performed using ImageJ software (version 1.54). Data analysis was performed using GraphPad Prism. The Kruskal–Wallis test was used to determine statistically significant differences between the medians of independent groups. The Dunn test was used as a post-hoc test to compare the experimental groups (bidirectional promoters), and Dunnett’s test was used to compare the experimental groups with the control group (single promoters).

## 3. Results

### 3.1. Promoter Design Rationale

**Figure 1:**
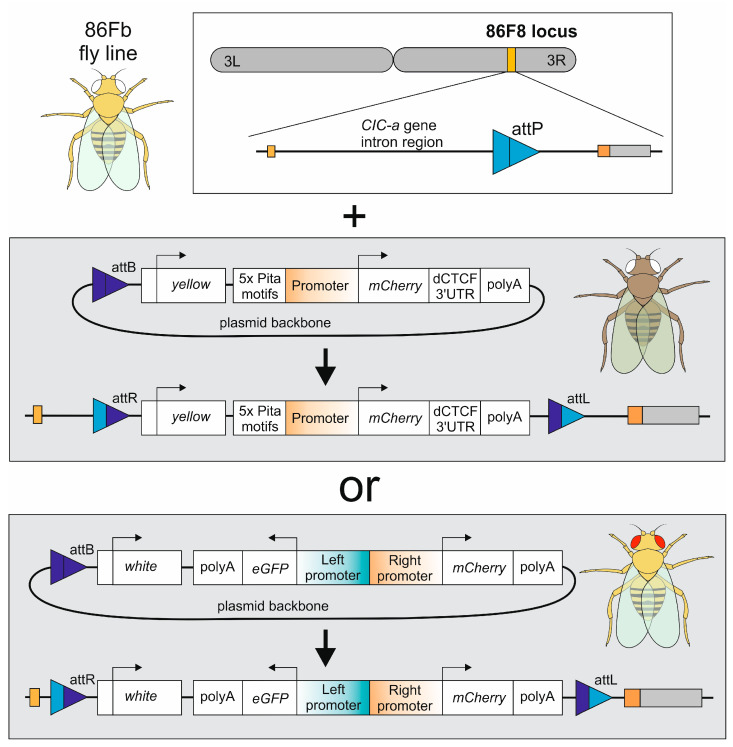
Scheme of fly genome modification approach and plasmids used.

The promoter strength was estimated by measuring the fluorescence intensity of mCherry or eGFP proteins as products of reporter genes under the control of the tested promoter. We used the *pBluSK* plasmid containing the promoter and protein-coding sequence for transfection of S2 cells and injection into *D. melanogaster* embryos to generate transgenic lines (Figure 1). In flies, the constructs were integrated at the 86F8 locus on the third chromosome. In S2 cells, the constructs were expressed transiently. The plasmids for single and bidirectional promoters differed in *white* and *yellow* reporters, as well as the presence/absence of additional Pita^×5^ sites and the last intron, exon, and the 3′UTR of the *dCTCF* gene. The Pita^×5^ sites were added as a boundary element to mitigate the impact of the surrounding chromatin, and the 3′ region of the *dCTCF* gene was inserted for intron-mediated enhancement [19] of transcription after integration into the Drosophila genome [20]. For S2 cells, fluorescence was quantitatively measured 2–3 days after transfection using flow cytometry. For the transgenic flies, total fluorescence was qualitatively imaged in embryos at stages 5 and 16 using confocal microscopy, and in third-instar larvae using fluorescence microscopy. On stage 5, the expression patter reflects the contribution of both the maternal and zygotic expression, while reflecting the zygotic expression only for stage 16.

In this study, we investigated the following single promoters (Table 1, Supplementary Figure S1). As a well-known strong promoter, we chose the constitutive promoter of the *ubiquitin-63E* gene (pUbi; Supplementary Figure S1). Constructs containing the *ubiquitin-63E* promoter have been used in *Drosophila* to express the Pita protein [20]. To test a translation-based approach to regulating protein levels, we designed a promoter of the same size as pUbi, but with the Kozak sequence changed from CAAAatgC to GTGAatgC (pUbi-K). The other four individual promoters investigated were those of transcription factor genes: *cp190* (*centrosomal protein 190 kD*), *pzg* (*putzig*), *ZIPIC* (*zinc-finger protein interacting with CP190*), and *CG10321* (Supplementary Figure S1). According to FlyBase (https://flybase.org), the transcript expression levels of all these genes are moderate to high in embryos, with the exception of *ZIPIC*, which has a low level.

To construct paired bidirectional promoters, we PCR-amplified (Table 1, Supplementary Figure S1) the *ubiquitin-63E* gene promoter (pUbi), its naturally adjacent promoter for the *trans-2,3-enoyl-CoA reductase* gene (pTecr), a short form of the *ribosomal protein L27a* promoter (139 bp) (mini-pRpl), and its modified long form (235 bp) with altered transcription factor binding sites (pRpl*; Supplementary Table S2). We prepared the weakened pRpl* promoter, which comprises substitution of the original DNA motifs for architectural proteins: motif 1 binding protein (M1BP) [21] and Optix-binding protein (Opbp) [22]. The new motif was incorporated in order to test the substitutability of architectural promoter-associated proteins. Promoters were arranged on opposite strands with their 5′ ends close to each other in the following combinations: eGFP::pTecr—pUbi::mCherry (naturally occurring combination; Supplementary Figure S1), eGFP::mini-pRpl—pUbi::mCherry (artificially combined), and eGFP::mini-pRpl—pRpl*::mCherry (artificially combined).

### 3.2. Analysis of the Strength of Single Promoters in the Model Systems in S2 Cells and Transgenic Drosophila

**Figure 2:**
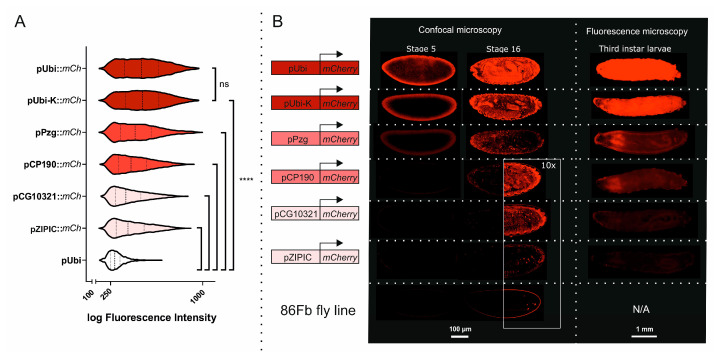
Estimation of single promoter strength: pUbi, pUbi-K, pPzg, pCP190, pZIPIC, and pCG10321. (**A**) Flow cytometry results of S2 cells transfected with plasmids comprising *mCherry* under different single promoters. (**B**) *D. melanogaster* embryos and third-instar larvae bearing constructs with *mCherry* under single promoters. The 86Fb fly line is used as a control of autofluorescence in embryos. Statistical significance: *p* > 0.05 (ns), *p* ≤ 0.0001 (****).

First, we analyzed the single promoters transiently in S2 cell culture to distinguish the promoters by strength and activity. According to flow cytometry results, among the tested constructs with a single promoter, plasmids containing the pUbi promoter showed the highest levels of mCherry fluorescence in S2 cells (Figure 2A). The transcriptional activity of the promoters was in the following order: pUbi > pPzg > pCP190 > pZIPIC > pCG10321. The strength of the pUbi and pUbi-K promoters did not differ significantly in the S2 cell model system.

The main goal of estimating the promoter strength is to classify the selected promoters as strong, medium, and weak, which will allow the level of ectopic expression to be modulated in the *Drosophila* model. Therefore, we conducted a comparative analysis of the strength of the promoters in embryos and larvae of the created transgenic lines. Fluorescence analysis was performed on embryos at developmental stages 5 and 16. Stage 5 corresponds to the completion of cellularization and the beginning of gastrulation. At this stage, a major wave of zygotic genome activation occurs, during which over 6000 genes are transcribed de novo [23]. Stage 16 marks the end of embryogenesis and the onset of larval hatching. The autofluorescence of the *Drosophila* egg layer is known, so the 86Fb fly line without a transgenic insertion was used as a control to differentiate between target eGFP and mCherry signals and embryo autofluorescence.

All single promoters (pUbi, pUbi-K, pPzg, pCP190, pZIPIC, and pCG10321) provided fluorescent signals that were sufficient for visualization in *D. melanogaster* embryos at both stages (Figure 2B). According to confocal microscopy results, the promoters could be ranked from strong to weak, with pUbi and pUbi-K being the strongest and pZIPIC being the weakest; the pPzg and pCP190 promoters fell in between these two extremes, while pCG10321 had a signal that was slightly detectable but not as strong as the other promoters. The signal strength increased from stage 5 to stage 16 for all promoters, indicating that transcription was enhanced during development under all conditions. In addition, we analyzed the promoter strength in *D. melanogaster* third-instar larvae (Figure 2B). The results of promoter transcription output, measured as the fluorescence reporter signal, were similar to those obtained in embryos. However, the difference in expression levels between moderate/weak promoters (pCP190, pCG10321, and pZIPIC) and strong promoters (pUbi and pUbi-K) was less distinct in larvae than in embryos.

### 3.3. Strength of Bidirectional Promoters in the Model Systems in S2 Cells and Transgenic Drosophila

**Figure 3:**
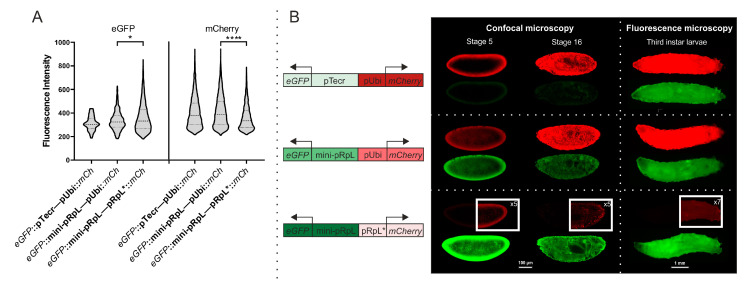
Estimation of bidirectional promoter strength: *eGFP*::pTecr—pUbi::*mCherry*, *eGFP*::mini-pRpl—pUbi::*mCherry* and *eGFP*::mini-pRpl—pRpl*::*mCherry*. (**A**) Flow cytometry results of S2 cells transfected with plasmids comprising *eGFP* and *mCherry* under bidirectional promoters. (**B**) *D. melanogaster* embryos and third-instar larvae bearing constructs with *eGFP* and *mCherry* under bidirectional promoters. Statistical significance: *p* ≤ 0.05 (*), *p* ≤ 0.0001 (****).

We compared a panel of bidirectional promoters in similar manners, transiently in S2 cells (Supplementary Figure S2) and in stable transgenic *Drosophila* lines at the embryonic and larval stages. Because it was not possible to directly compare the expression levels of eGFP and mCherry, constructs with bidirectional promoters were analyzed pairwise. No matter what the neighboring promoter was (pTecr or mini-pRpl), the expression of mCherry under the control of the pUbi was high (Figure 3A). When the pUbi was replaced with pRpl*, there was a decrease in mCherry expression if the mini-pRpl::GFP remained unchanged. Regardless of the neighboring promoter (pUbi or pRpl*), mini-pRpl provided stable expression of eGFP. Substitution of mini-pRpl with pTecr did not significantly affect the level of eGFP expression if the adjacent pUbi::*mCherry* remained unchanged. In general, the experiments in S2 cells do not indicate a position effect of the paired promoters, and each promoter appeared to function independently.

In transgenic fly lines all combinations of bidirectional promoters also produce fluorescent reporter signals that are robust enough for detection in embryos (Figure 3B). However, there were differences in their mode of joint action. *mCherry* under pUbi showed a high level of transcription. The strength of pUbi was slightly higher if pTecr::e*GFP* was adjacent, and lower when mini-pRpl::*eGFP* was adjacent. The substitution of pUbi with pRpl* significantly decreased the transcription of the *mCherry* gene, if the adjacent promoter, mini-pRpl, remained unchanged. *eGFP* under mini-pRpl produced a stable eGFP signal. The strength of mini-pRpl was significantly higher when pRpl*::*mCherry* was adjacent, while pUbi::mCherry strength was lower. The substitution of mini-pRpl for pTecr decreased the expression level of *eGFP* when the adjacent promoter pUbi remained unchanged. Thus, depending on the genome environment, the promoters showed different transcriptional outputs, with pUbi being significantly stronger than pRpl*, and mini-pRpl being stronger than pTecr. Comparisons of the remaining promoters could not be performed, as they have different reporter proteins. For bidirectional promoters, we observed increases in eGFP and mCherry synthesis from stages 5 to 16 in all promoters studied. This indicates a general increase in genome transcription during embryogenesis. The results in embryos and larvae were consistent for the *mCherry* gene, but there were differences in expression levels for *eGFP*. We observed similar levels of expression for pTecr and mini-pRpl promoters, which may be due to the specificity of their regulation during this stage of fly development.

## 4. Discussion

Promoters of transcription factors that express at constitutively low levels in cells may be a suitable source of weaker promoter sequences. Therefore, the promoters of genes *CG10321*, *CP190*, *putzig*, and *ZIPIC* were selected as potential alternatives to the well-known strong promoter of the *ubiquitin-63E* gene.

The length of the promoter sequences varies from around 1–2 kb (2001 bp for pUbi, 1191 bp for pCG10321, and 1065 bp for pCP190) to a more compact size (844 bp for pPzg and 266 bp for pZIPIC). The shortest promoter, pZIPIC, also exhibited the lowest transcriptional activity, indicating that the ZIPIC protein may be present at low levels in cells or its promoter activity may be additionally regulated.

We determined the strength of promoters in *Drosophila* S2 cells and transgenic flies at the embryonic and larval stages. The results obtained in the cells were generally comparable with those from the transgenic lines, with some minor differences. According to the results from the S2 cells, pZIPIC showed higher activity than pCG10321. However, in the transgenic model, pZIPIC proved to be the weakest promoter. It should be noted that *Drosophila* S2 cells are derived from a late-stage (20–24 h old) primary culture of *D. melanogaster* embryos and represent a heterogeneous population of differentiated cells. For practical use of promoter-containing constructs in genome integration, it is recommended to refer to data obtained in embryos and larvae. Overall, the generated single promoters could be classified as strong (pUbi and pUbi-K), moderate (pPzg and pCP190), and weak (pZIPIC and pCG10321), and therefore can be applied to various applications and expression levels.

In order to reduce biosynthesis through translational regulation, we mutated the Kozak sequence in a pUbi-containing construct. The most common Kozak sequence in *D. melanogaster* is CAAAatgG, with a predicted strength of 98% of the maximum possible score [11]. pUbi contains a CAAAatgC Kozak sequence with a predicted strength of 79% of maximum, but mutation of just three base pairs in the Kozak sequence to GTGAatgC decreases the predicted strength to only 25%. We tested this approach by designing a pUbi-K promoter with a CAAAatgC Kozak sequence. However, in S2 cells, the mutation did not lead to a statistically significant decrease in the mCherry signal, and there was only a slight qualitative decrease for *Drosophila* embryos and larvae.

We also constructed bidirectional promoters that can drive expression in both directions with varying efficiency. These pairs of bidirectional promoters allow for simultaneous expression at high/high (mini-pRpl—pUbi) or high/low (pTecr—pUbi and mini-pRpl—pRpl*) levels. The mini-pRpl—pRpl* bidirectional promoter is extremely short (386 bp) and may be a useful element for creating a compact cassette for the co-expression of two proteins with different levels of biosynthesis.

Regulatory elements located close to each other can affect the activity of each other, which is known as the “neighboring gene effect” and occurs when the insertion of a cassette at one locus reduces the expression of a nearby gene [24]. If two different promoters are artificially combined in one cassette, this neighboring gene effect may also occur. Our results show that depending on the sequence of the adjacent promoter, pUbi and mini-pRpl can change their transcription efficiency. Thus, if mini-pRpl, which is more active than pTerc, is adjacent to pUbi, then the pUbi strength decreases. Similarly, if a more active pUbi is adjacent to mini-pRpl instead of mini-pRpl*, then the mini-pRpl strength decreases. We propose that this interference between promoters could be a result of competition for shared regulatory proteins, such as transcription factors, the Mediator complex and RNA polymerase II, and their cofactors. To verify this model, further experiments are required.

## 5. Conclusions

Promoter strength plays a critical role in regulating protein expression. In order to facilitate the more rational selection of promoters for ectopic gene expression in *D. melanogaster*, we have generated a set of constructs consisting of promoter sequences arranged singly and in pairs, directed oppositely. Individual promoters can be classified as strong, medium, and weak, each with varying levels of efficiency for transcription. Among these, weak promoters, which have been underrepresented in the literature, are of particular interest. We have also generated bidirectional promoters that enable simultaneous transcription with either high/high or high/low efficiency. Due to their compact design, these constructs hold particular promise for research that involves the synthesis of two proteins at different levels of transcription in a single cassette. Our results also suggest a competition between bidirectional promoters for transcription machinery, but this hypothesis requires further investigation.

## Figures and Tables

**Table 1:** Promoters used in the study. Promoter sequences include ATG start codons.

Name	*D. melanogaster* Gene	Modification	Size (bp)
Single promoters			
pUbi	*ubiquitin-63E*	-	2001
pUbi-K	*ubiquitin-63E*	CAAAatgC Kozak seq. is mutated to GTGAatgC	2001
pCG10321	*CG10321*	-	1191
pCP190	*centrosomal protein 190kD*	-	1065
pPzg	*putzig*	-	844
pZipic	*ZIPIC*	-	266
Bidirectional promoters			
pTecr—pUbi	*Trans-2,3-enoyl-CoA reductase*/*ubiquitin-63E*	-	1805
mini-pRpl—pUbi	*ribosomal protein L27a/ubiquitin-63E*	-	1949
mini-pRpl—pRpl*	ribosomal protein L27a/ribosomal protein L27a*	*: M1BP (cggtcacactg) and Opbp (caaccgcagccaactt) motifs of mini-pRpl27a were replaced with sequence tatcgata-tttt-tatcgata	386

## Data Availability

Data available on request from the authors.

## Supplementary Materials

The following supporting information can be downloaded at www.mdpi.com/article/10.3390/genes16010003/s1, Table S1: Additional fragments used for pBlueSK plasmid modification; Table S2: Promoter sequences used for PCR amplification; Figure S1: Location of the tested promoter sequences in the *D. melanogaster* genome. Figure S2: S2 cells transfected with plasmids comprising *eGFP* and *mCherry* under bidirectional promoters. Images were acquired on a Nikon Ti fluorescence microscope with Nikon Plan Fluor 10×/0.3 objective.
