# Supplementary material for: Construction of Promoter Elements for Strong, Moderate, and Weak Gene Expression in Drosophila melanogaster
Source: Genes (Basel). 2024 Dec 24;16(1):3. doi: 10.3390/genes16010003 (PMC11764520; doi:10.3390/genes16010003)
Supplement: Supplementary file 1 [file genes-16-00003-s001.zip › genes-3341312-supplementary.pdf]

Supplementary materials

Supplementary Table 1. Additional fragments used for pSK plasmid modification.

<table>
<tr><td>5x Pita binding motifs</td><td>ctttagccaagacgcgaacccgaatccgaaa|ctttagccaagacgcgaacccgaatccgaaa|ctttagccaagacgcgaacccgaatccgaaa|cttagccaagacgcgaacccgaatccgaaa|ctttagccaagacgcgaacccgaatccgaag</td></tr>
<tr><td>dCTCF 3’UTR</td><td>ttggtttcgaggttagttgcaaaaactttagctggcatttgttccattactcaaaaatttggttttcatgcaggacgacgatgattatgaggagtatggcgatggggaaaacgaggtcgatggcgcttcccaggagtttctgcagctgatggatatgattgagcaggactcttagtctcctgaaaacaaaagctaatggataagtaattatgactaacttatgtacataccaagcatacaataacgtatttctacgttacattgttttctatttaaatacacatttaaaacagttaggaaacctatttttactaggttaagaaatggatattaaaaatgtattctttctaaatgaatgtataattctggataagcagttaacatgccgatatggcgactatttgtaaatgatccccgacacttaaactttagttgacaacttggcat</td></tr>
</table>

Supplementary Table 2. Promoter sequences used for PCR amplification.

Promoter name	Promoter sequence
pUbi	tgtcgccggaacgcagcgacagggattccaatgtgtccgtatcctttaggcttttgcccttcagttccagacgaagcgactggcgattcgcgtgtggggtctgcttcagggtcttgtgaattagggcgcgcagatcgccgatgggcgtggcgccggagggcaccttcaccttgccgtacggcttgctgttcttcgcgttcaaaatctccagctccattttgctttcggtgcgcttgcaatcagtactgtccaaaatcgaaaatcgccgaaccgtagtgtgaccgtgcggggctctgcgaaaataaacttttttaggtatatggccacacacggggaaagcacagtggattatatgtattaatatattatccaggttttcattacttatccagatgtaagcccacttaaagcgatttaacaattatttgccgaaagagtataaacaaattttacttaaaaatggattaagaaaagcttgtgtaagattatgcgcagcgttgccagatagctccatttaaaacacttcaaaaacaataagttttgaaaatatatacataaatagcagtcgttgccgcaacgctcaacacatcacacttttaaaacaccctttacctacacagaattactttttaaatttccagtcaagctgcgagtttcaaaattatagccggtagagaagacagtgctatttcaaaagcaaactaacaagggtcttaaattccaaaacaccaatcctaacaagccttggacttttgtaagtttagatcaaaggtggcattgcattcaatgtcatggtaagaagtaggtcgtctaggtagaaatcctcattcagccggtcaagtcagtacgagaaaggtctcaatttgaaattgtcttaaaaatattttattgttttgtactgtggtgagtttaaacgaaaaacacaaaaaaaaagtgatacatagaaatcataaaaaattttaatacaaggtattcgtacgtatcaaaaacatttcggcacaatttttttctctgtactaaagtgttacgaacactacggtattttttagtgattttcaacggacaccgaaggtatataaacagcgttcgcgaacggtcgccttcaaaaccaattgacatttgcagcagcaagtacaagcagaaagtaaagcgcaatcagcgaaaaatttatacttaattgttggtgattaaagtacaattaaaagaacattctcgaaagtcacaagaaacgtaagtttttaactcgctgttaccaattagtaataagagcaacaagacgttgagtaatttcaagaaaaactgcatttcaaggtctttgttcggccatttttttttattcaacgctctacgtaattacaaaataagaaattggcagccacgcatctcgttttcccaatgaattggcatcaaaacgcaaacaaatctataaataaaacttacgtgttgattttcgccaagatttattggcaaattgtgaaattcgcagtgacgtatttgaaaattcgagaaatcacatacgcactcgagcatttgtgtgcatgttattagttagttgtttagttaattgaagtattttaccaacgaaatccacttatttttagctgaaatagagtaggttgcttaaacaaagccacgtctgaaaatttcttattgcttgtagttgtgacgtcaccatatacacacaaaataatgtgtatgcatgcatttcagctgtgtatacatacatgcacacactcgcaacacgaaaacgatgacgaagcaacggaacaaaggtttctcaactaccctttgttccctgtttcttcgctttcctttgttccaatattcgtagagggttaataggggtttctcaacaaagttggcgtcgataaataagtttcccatttttattccccagccagaaagttagtttcaatagttttgtaatttcaacgaaactcgtctgatttcgtactaattttccacatctctattttcttcccgcagaataatccaaaatg
pUbi-K	tgtcgccggaacgcagcgacagggattccaatgtgtccgtatcctttaggcttttgcccttcagttccagacgaagcgactggcgattcgcgtgtggggtctgcttcagggtcttgtgaattagggcgcgcagatcgccgatgggcgtggcgccggagggcaccttcaccttgccgtacggcttgctgttcttcgcgttcaaaatctccagctccattttgctttcggtgcgcttgcaatcagtactgtccaaaatcgaaaatcgccgaaccgtagtgtgaccgtgcggggctctgcgaaaataaacttttttaggtatatggccacacacggggaaagcacagtggattatatgtattaatatattatccaggttttcattacttatccagatgtaagcccacttaaagcgatttaacaattatttgccgaaagagtataaacaaattttacttaaaaatggattaagaaaagcttgtgtaagattatgcgcagcgttgccagatagctccatttaaaacacttcaaaaacaataagttttgaaaatatatacataaatagcagtcgttgccgcaacgctcaacacatcacacttttaaaacaccctttacctacacagaattactttttaaatttccagtcaagctgcgagtttcaaaattatagccggtagagaagacagtgctatttcaaaagcaaactaacaagggtcttaaattccaaaacaccaatcctaacaagccttggacttttgtaagtttagatcaaaggtggcattgcattcaatgtcatggtaagaagtaggtcgtctaggtagaaatcctcattcagccggtcaagtcagtacgagaaaggtctcaatttgaaattgtcttaaaaatattttattgttttgtactgtggtgagtttaaacgaaaaacacaaaaaaaaagtgatacatagaaatcataaaaaattttaatacaaggtattcgtacgtatcaaaaacatttcggcacaatttttttctctgtactaaagtgttacgaacactacggtattttttagtgattttcaacggacaccgaaggtatataaacagcgttcgcgaacggtcgccttcaaaaccaattgacatttgcagcagcaagtacaagcagaaagtaaagcgcaatcagcgaaaaatttatacttaattgttggtgattaaagtacaattaaaagaacattctcgaaagtcacaagaaacgtaagtttttaactcgctgttaccaattagtaataagagcaacaagacgttgagtaatttcaagaaaaactgcatttcaaggtctttgttcggccatttttttttattcaacgctctacgtaattacaaaataagaaattggcagccacgcatctcgttttcccaatgaattggcatcaaaacgcaaacaaatctataaataaaacttacgtgttgattttcgccaagatttattggcaaattgtgaaattcgcagtgacgtatttgaaaattcgagaaatcacatacgcactcgagcatttgtgtgcatgttattagttagttgtttagttaattgaagtattttaccaacgaaatccacttatttttagctgaaatagagtaggttgcttaaacaaagccacgtctgaaaatttcttattgcttgtagttgtgacgtcaccatatacacacaaaataatgtgtatgcatgcatttcagctgtgtatacatacatgcacacactcgcaacacgaaaacgatgacgaagcaacggaacaaaggtttctcaactaccctttgttccctgtttcttcgctttcctttgttccaatattcgtagagggttaataggggtttctcaacaaagttggcgtcgataaataagtttcccatttttattccccagccagaaagttagtttcaatagttttgtaatttcaacgaaactcgtctgatttcgtactaattttccacatctctattttcttcccgcagaataatccaaaatg
pCP190	gaatcaaagtgtttttaacgtctcgtggtggccaagccgacgacccgttttatatattttgattgcaaagaagggtggcgcaagaaatgtgacacattacgatgtcacttttcaaaatttcaatagtttaagtaaatgtattttgttttcaactgcaaaacaatatttattaaagtattttaagtatatgtattgtgtttagtaagatttagcttaaactgcaataaccttaaagtatatcatattcaaatatttttagacttaacg

	attaacttcatctgcgtttacgtcaccttaaaaatgttaagcatgagactgaggttatcgcatggctatcgatagctgccgagcgc<br>aaccctgtcacgccaacgatgaatggaaaagaatatggcgacctgactctggaaaaaaattataatttgtccggcaaaagtag<br>tgtgcaattcaaagaaatgtatgtattaaaagtgcaattgtgcggcgattgtctgaaaaaagtacattcaatcaaaggacacgca<br>gtaaacaagccgctgaggtcaaagtgatttgtgcgtgccccaaaaaaaccattgttgtgtgaagtggacaggcgcgccaaaa<br>gggctcaaaaaatgccacccaccgaaagatgccaaagttggacttacaagaaaacattgcagaattgcggacaatgaacaa<br>ttactgataaataatacaagaagcctggcgacgcgcacatcgcacacatacgcccgcgaaactgtcacacgtacacaggcg<br>tatatcgatatttgcgagttgcatttcgcgccgtggcggcagagcaaaataaaccgaataacccaaaatcaacgggcgtctatt<br>tgaaactaacagcggaatataccatacttactaattgttattctttcttgttttacccattcacctgcgtcccatcccaatacgcttttt<br>ccgactccttaccccctgcgtgagtacagttgcgccctgaagccagagcaagcgaaaccacggggaggctaacgataagga<br>tacggaaacggagagcacggccactagccaagcacacagcaatatg
pPzg	attagtgatgttggcctttatcactgcgtaaatgcaagtatttaatgcttacctcacaggttgggtatgccaaatatttacgaggag<br>aatatttgtgaatttaaagtgggcgggcaaaggcgccctctactgtctacaaaacgcactgttaattttgacattgcatgatagg<br>aatgacgctgagcctttttcaagacaaaatgtgctaattatttaccttttacaaatacaagcacaacaagtaagtatttatttgtattt<br>aaccactcagcaatcgcaataagaaaaataaatgctttattatttcaagtaaataattacgttatcgtaatgaacggcaacgctgt<br>gagattttctctgtctgaccatggttgacctcccaggcagccgccatttccgtggcgtgcgagtgaaacgactagcccatgtttt<br>cccgccgtgccagggcagcgagcatgctaaagagcgagacgaccatacgcgtccgtcgagaactgctacagcacatacg<br>catttttaaccctaaaaaatacagcaccgagtctttagtacagtcaaagtgacgccgcgacaactcagaacgaaaccgaaaat<br>taaaaacggaagagattgttgttcctggtgtccaaaaaagatagattgtttcgttcgagtggctcatccgtacccgatcgataca<br>taaatcccagcaagctgccaaaccggagtgcagaagtgtagtttgtcgtaatcgccgattaatattgaaaggtagcggcttgc<br>agttgcgcgtgtagcgcccgcacaaaccgaataacaccatcactacggcgtgctcacctgaatccaaaacgccacgaaatg
pZipic	ttcctaactgccctgttggcaatttataatacaacaaaaaattagtaaatacatggctacatatcgatttgtttttcgctgggtgtta<br>caaattttcagcttttgattttgagtttcagatctgctttgttggttagcagccaaagttcaaatttggaaattgaaccagatcacatc<br>gtcgataaccgatagctgtgcaatcggagccttcttcagggcacggtcacactgccccgaaagtgtaaataaatttcaatcaa<br>atatg
pCG10321	caggatcaacaggtgttttcagttcgaaatgtcagtttaaaaaacgagttggcagctcaattgctatgtcctcgaattaaaatttgt<br>gtgttcgtttttgggttagatttattaaaaatttcggcttgaaaatgttaatgtgagtcaaaaaactgttgtgtgtttcattgaatatgt<br>aaagaaatccccctggtatgaacggcaaggaatggctctaaaatcgagaaattaaagagacttgtgagctgaaggacccacat<br>caaaaacctaagttgtaaatagagaaaaggtaagtttcaatggaatttcatgccttaaacataagaagttttaagtattaaaaaga<br>aatctagaaatctgaggaaataaacaaaatgatttccgaccggcatgcgttagggatttctgtctttataccacgttggcagcgc<br>cattcacaacacgcctatcatctcactcacactcgcacacagagaaagacagatagaggcagaagttcagttcatcaattcatt<br>catctcatggaagaaccttattgataacgtcgccagagatagctagtgatttcattcgatcagggtcccgaaagacccactggt<br>gtgcacttgttttggttttgaagttcgcatctctgggaacgtgtgtgtttcgccctgaaccacaaatatgtgagttccgaatgcgat<br>ttgtgtgacgcggccatatttaaagagtttttgtgttgaatgtggggcgagctgcgaaaattacataaatacagcgcaggtgag<br>ttcatttttgttgcgggaacccggtcgaaaggtgctctaagtgcgggtgcaagtgaatcgaaaacaatatttacccaattcaagt<br>cgggaattatgtatttgtttcgtttttgggcctaaaaatcgcatttttcgctcacaccaacaccaagatggtaaattgtgtataaca<br>atatggctgtctctgtctggcacaagagctaccagacgtgtcgacagtccctgttattaacagcgggttagcaggtcattttaca<br>acagatgaaattattctgaattttaattgaccatttttggcgggcaaatcataacaaacctcgtactcatgcacctttcggggatc<br>cgctcattctaatggttattttaaattaaaaaaaactggcccattgcagcctcccccctaggaacacgaagtgatg
pTecr — pUbi	catatcgaattcggcggatcctttgctttcggtgcgcttgcaatcagtactgtccaaaatcgaaaatcgccgaaccgtagtgtga<br>ccgtgcggggctctgcgaaaataaactttttaggtatatggccacacacggggaaagcacagtggattatatgtattaatatat<br>tatccaggttttcattacttatccagatgtaagcccacttaaagcgatttaacaattatttgccgaaagagtataaacaaattttactt<br>aaaaatggattaagaaaagcttgtgtaagattatgcgcagcgttgccagatagctccatttaaaacacttcaaaaacaataagtt<br>ttgaaaatatatacataaatagcagtcgttgccgcaacgctcaacacatcacacttttaaaacaccctttacctacacagaattac<br>tttttaaatttccagtcaagctgcgagtttcaaaattatagccggtagagaagacagtgctatttcaaaagcaaactaacaaggg<br>tcttaaattccaaaacaccaatcctaacaagccttggacttttgtaagtttagatcaaaggtggcattgcattcaatgtcatggtaa<br>gaagtaggtcgtctaggtagaaatcctcattcagccggtcaagtcagtacgagaaaggtctcaatttgaaattgtcttaaaaata<br>ttttattgttttgtactgtggtgagtttaaacgaaaaacacaaaaaaaaaagtgatacatagaaatcataaaaaattttaatacaagg<br>tattcgtacgtatcaaaaacatttcggcacaattttttttctctgtactaaagtgttacgaacactacggtatttttttagtgattttcaac<br>ggacaccgaaggtatataaacagcgttcgcgaacggtcgccttcaaaaccaattgacatttgcagcagcaagtacaagcag<br>aaagtaaagcgcaatcagcgaaaaatttatacttaattgttggtgattaaagtacaattaaaagaacattctcgaaagtcacaag<br>aaacgtaagtttttaactcgctgttaccaattagtaataagagcaacaagacgttgagtaatttcaagaaaaactgcatttcaagg

	tctttgttcggccatttttttttattcaacgctctacgtaattacaaaataagaaattggcagccacgcatctcgttttcccaatgaatt<br>ggcatcaaaacgcaaacaaatctataaataaaacttacgtgttgattttcgccaagatttattggcaaattgtgaaattcgcagtg<br>acgtatttgaaaattcgagaaatcacatacgcactcgagcatttgtgtgcatgttattagttagttgtttagttaattgaagtattttac<br>caacgaaatccacttatttttagctgaaatagagtaggttgcttaaacaaagccacgtctgaaaatttcttattgcttgtagttgtga<br>cgtcaccatatacacacaaaataatgtgtatgcatgcatttcagctgtgtatacatacatgcacacactcgcaacacgaaaacg<br>atgacgaagcaacggaacaaaggtttctcaactaccctttgttccctgtttcttcgctttcctttgttccaatattcgtagagggtta<br>ataggggtttctcaacaaagttggcgtcgataaataagtttcccatttttattccccagccagaaagttagtttcaatagttttgtaat<br>ttcaacgaaactcgtctgatttcgtactaattttccacatctctattttcttcccgcagaataatccaaaatg
mini-pRpL — pUbi	catatcgaattcgcggccgcaacgaattcacgtgcaagatgcgcttcaattgacagccgcaagcaaaaaggaagttgagtcg<br>atgtacttagctgctttaaaattgtaggaaagaaagaagcgaagttggctgcggttgtagcagtgtgaccgcccggcggatcc<br>tttgctttcggtgcgcttgcaatcagtactgtccaaaatcgaaaatcgccgaaccgtagtgtgaccgtgcggggctctgcgaaa<br>ataaactttttttaggtatatggccacacacggggaaagcacagtggattatatgtattaatatattatccaggttttcattacttatcc<br>agatgtaagcccacttaaagcgatttaacaattatttgccgaaagagtataaacaaattttacttaaaaatggattaagaaaagct<br>tgtgtaagattatgcgcagcgttgccagatagctccatttaaaacacttcaaaaacaataagttttgaaaatatatacataaatag<br>cagtcgttgccgcaacgctcaacacatcacacttttaaaacaccctttacctacacagaattactttttaaatttccagtcaagctg<br>cgagtttcaaaattatagccggtagagaagacagtgctatttcaaaagcaaactaacaagggtcttaaattccaaaacaccaat<br>cctaacaagccttggacttttgtaagtttagatcaaaggtggcattgcattcaatgtcatggtaagaagtaggtcgtctaggtaga<br>aatcctcattcagccggtcaagtcagtacgagaaaggtctcaatttgaaattgtcttaaaaatattttattgttttgtactgtggtga<br>gtttaaacgaaaaacacaaaaaaaaaagtgatacatagaaatcataaaaaaattttaatacaaggtattcgtacgtatcaaaaacat<br>ttcggcacaattttttttctctgtactaaagtgttacgaacactacggtattttttagtgattttcaacggacaccgaaggtatataaa<br>cagcgttcgcgaacggtcgccttcaaaaccaattgacatttgcagcagcaagtacaagcagaaagtaaagcgcaatcagcg<br>aaaaatttatacttaattgttggtgattaaagtacaattaaaagaacattctcgaaagtcacaagaaacgtaagtttttaactcgct<br>gttaccaattagtaataagagcaacaagacgttgagtaatttcaagaaaaactgcatttcaaggtctttgttcggccatttttttttat<br>tcaacgctctacgtaattacaaaataagaaattggcagccacgcatctcgttttcccaatgaattggcatcaaaacgcaaacaa<br>atctataaataaaacttacgtgttgattttcgccaagatttattggcaaattgtgaaattcgcagtgacgtatttgaaaattcgagaa<br>atcacatacgcactcgagcatttgtgtgcatgttattagttagttgtttagttaattgaagtattttaccaacgaaatccacttattttta<br>gctgaaatagagtaggttgcttaaacaaagccacgtctgaaaatttcttattgcttgtagttgtgacgtcaccatatacacacaaa<br>ataatgtgtatgcatgcatttcagctgtgtatacatacatgcacacactcgcaacacgaaaacgatgacgaagcaacggaaca<br>aaggtttctcaactaccctttgttccctgtttcttcgctttcctttgttccaatattcgtagagggttaataggggtttctcaacaaagt<br>tggcgtcgataaataagtttcccatttttattccccagccagaaagttagtttcaatagttttgtaatttcaacgaaactcgtctgattt<br>cgtactaattttccacatctctattttcttcccgcagaataatccaaaatg
mini-pRpL — pRpL*	catatcgaattcacgtgcaagatgcgcttcaattgacagccgcaagcaaaaaggaagttgagtcgatgtacttagctgctttaa<br>aattgtaggaaagaaagaagcgaagttggctgcggttgtagcagtgtgaccgcccggcggatccataatgcatatagactta<br>aatttatcttaaatataatggtgtgtacgagatatttttttcagactaaaatggtagttaggggtatatgttttctgtccggatggtattt<br>cgtatttttttgttatcgccgccgggtatcgatatttcttttatcgatacgcttctttctttcctacaattttaaagcagctaagtacatcg<br>actcaacttcctttttgcttgcggctgtcaattgaaatg<br><br>*Replaced sites are underlined*

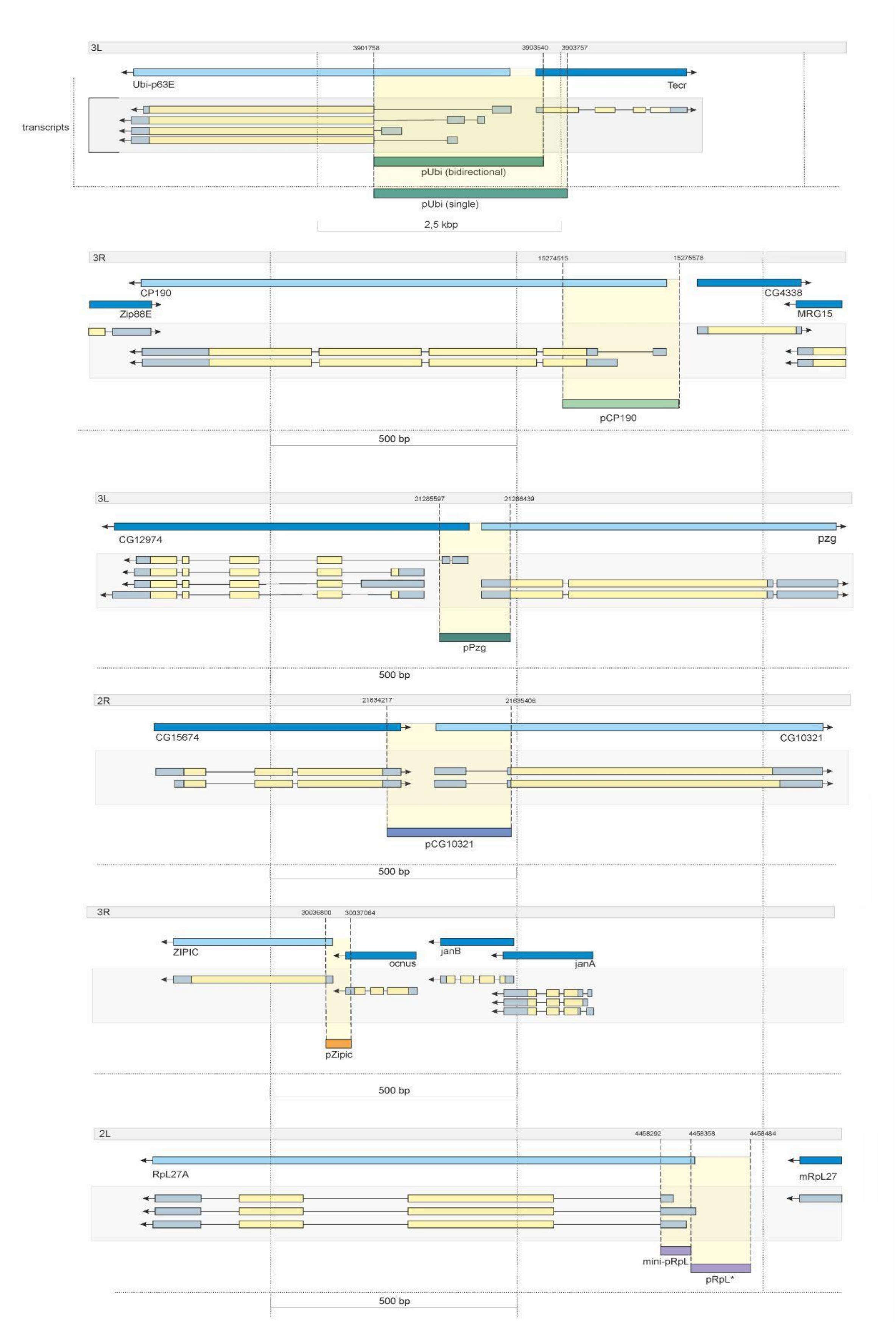

Supplementary Figure 1. Location of the tested promoter sequences in the *D. melanogaster* genome.

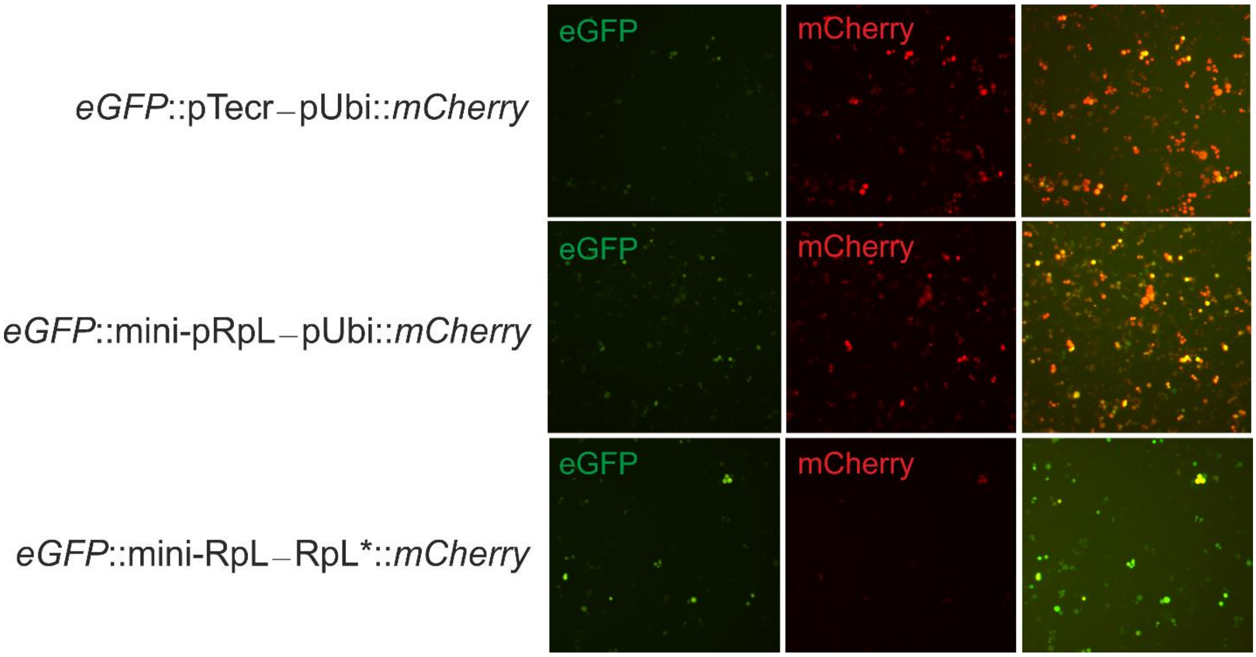

Supplementary Figure 2. S2 cells transfected with plasmids comprising *eGFP* and *mCherry* under bidirectional promoters. Images were acquired on a Nikon Ti fluorescence microscope with Nikon Plan Fluor 10X/0.3 objective.
